# Relationship Between Psychological Distress, Burnout and Work Engagement in Workers During the COVID-19 Pandemic: A Systematic Review

**DOI:** 10.3389/ijph.2022.1605605

**Published:** 2023-01-05

**Authors:** Ingrid Adanaqué-Bravo, Kenny Escobar-Segovia, Juan Gómez-Salgado, Juan Jesús García-Iglesias, Javier Fagundo-Rivera, Carlos Ruiz-Frutos

**Affiliations:** ^1^ Faculty of Engineering in Mechanics and Production Sciences, Escuela Superior Politécnica del Litoral, Guayaquil, Ecuador; ^2^ Faculty of Engineering in Earth Sciences, Escuela Superior Politécnica del Litoral, Guayaquil, Ecuador; ^3^ Department of Sociology, Social Work and Public Health, Faculty of Labour Sciences, University of Huelva, Huelva, Spain; ^4^ Safety and Health Postgraduate Programme, Universidad Espíritu Santo, Guayaquil, Ecuador; ^5^ Centro Universitario de Enfermería Cruz Roja, Universidad de Sevilla, Sevilla, Spain

**Keywords:** burnout, COVID–19, psychological distress, stress, work engagement

## Abstract

**Objective:** The psychological distress that the COVID-19 pandemic has produced has generated negative effects on workers, and in one way or another this has affected their work engagement within companies. The aim of this research was to assess the relationship between psychological distress, burnout and work engagement in workers during the COVID-19 pandemic.

**Methods:** A systematic review was carried out following the PRISMA methodology, taking articles from the Scopus, Pubmed, and Web of Science databases from the beginning of the pandemic until November 2022. The methodological quality was assessed using the Joanna Briggs Institute (JBI) critical appraisal tools for non-randomised studies.

**Results:** 24 articles were selected. All the articles found an association between psychological distress, burnout or other factors and work engagement.

**Conclusion:** The COVID-19 pandemic has had an impact on work engagement and a negative relationship with psychological distress and burnout, hence the importance of companies taking measures to minimise levels of psychological distress and burnout.

## Introduction

As the pandemic has progressed, some studies have dealt with its negative effects, specifically concerning the psychological impact it has had on workers from different areas worldwide (1–4), as well as the impact that certain variables such as the type of work, organisational climate, among others, may have on workers (5). According to Matziari et al. (6), burnout and work engagement are psychological reactions that are developed when individual characteristics interact with job characteristics, and which are based on the Job Demands Resources Model (7). One of these variables is work engagement, which is defined as the relationship between work and the conviction of being able to perform it effectively. This variable in turn involves a series of dimensions such as vigour (high energy level), dedication (identification with the work), and absorption (concentration on the work) (8). In this sense, work engagement is an important part of the productivity development of companies, where high standards of job satisfaction, adequate satisfaction with family life, and sufficient self-perceived health are required (9).

The COVID-19 pandemic has in one way or another affected the work engagement of workers in general, as determined by studies carried out in different countries and work areas (10–12). Likewise, other factors may also influence work engagement such as sickness presenteeism (SP) (defined as continuing to perform duties in the workplace despite working below full capacity due to illness) (13), meaningful work (10), sleep quality (14), emotional intelligence (15), etc.

It should be noted that, during the pandemic, companies had to find alternative ways of doing work, in some cases shifting to teleworking or even modifying working hours. In other companies, they switched to mixed types of hiring, i.e., people working from home and people who had to go to the office despite the confinement that was implemented in most countries, and this situation could have also affected engagement (16–18).

Psychological distress can negatively affect work engagement. In a study on non-healthcare workers, it was found that there were statistically significant differences between people with and without psychological distress. However, workers with the highest percentages of psychological distress showed low levels for the subdimensions of work engagement (vigour, dedication, and absorption) (11). It should be taken into account that chronic interpersonal work-related stress or stressors can also trigger emotional exhaustion, cynicism, or detachment from work, which may lead to chronic stress or burnout (19).

Burnout can be a predisposing indicator for the development of social and mental disorders, especially important in those people who have a certain predisposition for psychological disorders, taking medications or committing suicide attempts, among others (20). Any change can generate a stressful situation, even increased in a context of uncertainty and complexity of approach. This psychological distress, persisted over time, can lead to burnout. This can be a major public health problem in which work, family, and society interrelate and in which an interdisciplinary and community approach is needed. In fact, during COVID-19, many workers have been particularly exposed to the disease, some have lost their jobs, and many have seen their working conditions changed, with the consequent mental impact that this entails (21). For example, in a longitudinal study conducted on a sample of 1,308 Finnish workers, an increase in psychological distress and technostress was found during the COVID-19 crisis especially derived from a change in the conditions of their work, being especially greater in young women (22). Similarly, some occupational groups such as cleaners or healthcare workers have seen their workload increased in a context of greater exposition to COVID-19, in order to address emerging needs in society (23). Other professional groups have had to modify their way of working (teleworking, change of destinations and functions, etc.) and others have been forced into unemployment or temporary unemployment (24).

The aim of this research was to assess the relationship between psychological distress, burnout and work engagement in workers during the COVID-19 pandemic through a systematic review based on the PRISMA methodology.

## Methods

### Study Design

A systematic review was conducted following the guidelines of the PRISMA (Preferred Reporting Items for Systematic Reviews and Meta-Analyses) statement (25). To this end, the authors relied on a protocol to carry out this systematic review, which was registered in the International Prospective Register for Systematic Reviews (PROSPERO) of the University of York, with identification code CRD42022350318.

### Search Strategy

The search was carried out in the Pubmed, Scopus, and Web of Science electronic databases, based on the keywords from the research question generated by following the PICOT strategy (Table 1).

**TABLE 1:** PICOT format (COVID-19, Ecuador, 2020–2022).

Population	Healthcare and non-healthcare workers
Intervention	Level of stress and/or burnout
Comparator	Work engagement
Outcomes/Results	Levels by type of work, differences between healthcare staff/non-healthcare staff, differences between frontline staff vs. non-face-to-face staff
Time	During the COVID-19 pandemic
Research question	How does stress and/or burnout affect work engagement in workers during the COVID-19 pandemic?

The Medical Subject Headings (MeSH) descriptors used were: Psychological Distress; Burnout; Work engagement; and COVID-19. In order to enlarge the scope of the search, synonymous terms were used to complete the search based on the MeSH descriptors, linked using the Boolean operators AND and OR (Table 2).

**TABLE 2:** Search terms (COVID-19, Ecuador, 2020–2022).

MeSH	Terms
Psychological Distress	Psychological Distress, Emotional Distress, Emotional Stress
Professional Burnout	Professional Burnout, Occupational Burnout, Career Burnout
Work Engagement	Work Engagement, Employee Engagement, Staff Engagement, Workplace Engagement, Employee Participation, Worker Participation, Staff Participation
COVID-19	COVID-19, 2019-nCoV Infection, SARS-CoV-2 Infection, 2019 Novel Coronavirus Disease, COVID-19 Virus Infection, Coronavirus Disease 2019, 2019-nCoV Disease, COVID-19 Pandemic

Table 3 shows the search strategy used, carried out on 5 November 2022, for each of the databases during the search process.

**TABLE 3:** Search strategy and databases (COVID-19, Ecuador, 2020–2022).

Database	Search strategy	Search date	Results
Pubmed	((stress*[Title/Abstract] OR burnout [Title/Abstract]) AND (COVID-19 [Title/Abstract])) AND (Work engagement [Title/Abstract])	5 November 2022	63
Scopus	(TITLE-ABS-KEY (stress* OR burnout) AND TITLE-ABS-KEY (COVID-19) AND TITLE-ABS-KEY (work AND engagement))	5 November 2022	257
Web Of Science	stress* OR burnout (Topic) and COVID-19 (Topic) and Work engagement (Topic)	5 November 2022	384
Total			704

### Selection Criteria

The following selection criteria were used to select the articles:

#### Inclusion Criteria

- Original articles published in English and Spanish.
- Type: original articles.
- Articles measuring any of the following values and/or effects: level of burnout, level of depression, level of stress and work engagement, number of cases of professionals with depression, stress and/or anxiety, comparison of levels before vs. during the COVID-19 pandemic, and comparison by country/type of profession/service.

#### Exclusion Criteria

- Studies written in a language other than English and Spanish.
- Population: unemployed people.
- Studies of low scientific-technical quality after applying the quality assessment tool.
- Articles that did not answer the research question and were not related to the objective of the review.
- Typology: opinion articles, editorials and letters to the editor, systematic reviews, short communications, and case reports.

### Data Collection and Extraction

Once the data extraction was completed, two authors were in charge of the selection process by independently following the established inclusion and exclusion criteria, eliminating duplicate studies, and selecting articles that could be included after reading the abstract and title. Subsequently, the same two authors reviewed the full text of the studies which were potentially eligible for inclusion in the review, reaching a consensus; discrepancies were resolved by a third author.

### Assessment of Methodological Quality

After selecting the articles for the review, two reviewers independently determined the methodological quality of the selected studies using the Joanna Briggs Institute (JBI) of the University of Adelaide, Australia, critical appraisal tools for non-randomised studies (26). This allowed assessing the methodological quality of the studies and determining the extent to which a study had avoided or minimised the risks of bias in its design, conduct, and/or analysis. The cross-sectional quantitative study versions were used (27) (8 items) with a cut-off point of 6 for inclusion in this review (Tables 4, 5).

**TABLE 4:** Study scoring according to the JBI tools (COVID-19, Ecuador, 2020–2022).

Study	JBI	The participants and the environment are described in detail	Inclusion criteria are clearly defined	Exposure was measured in a valid and reliable way	The criterion used to measure the condition was objective	Confounding factors were identified	Strategies for dealing with confounding factors	Validly and reliably measured results	Appropriate statistical analysis was used
(42)	6/8	YES	YES	YES	YES	NA	NA	YES	YES
(10)	6/8	YES	YES	YES	YES	NA	NA	YES	YES
(43)	6/8	YES	YES	YES	YES	NA	NA	YES	YES
(44)	6/8	YES	YES	YES	YES	NA	NA	YES	YES
(45)	6/8	YES	YES	YES	YES	NA	NA	YES	YES
(46)	6/8	YES	YES	YES	YES	NA	NA	YES	YES
(11)	6/8	YES	YES	YES	YES	NA	NA	YES	YES
(21)	6/8	YES	YES	YES	YES	NA	NA	YES	YES
(47)	6/8	YES	YES	YES	YES	NA	NA	YES	YES
(29)	6/8	YES	YES	YES	YES	NA	NA	YES	YES
(30)	6/8	YES	YES	YES	YES	NA	NA	YES	YES
(15)	6/8	YES	YES	YES	YES	NA	NA	YES	YES
(5)	6/8	YES	YES	YES	YES	NA	NA	YES	YES
(31)	6/8	YES	YES	YES	YES	NA	NA	YES	YES
(32)	6/8	YES	YES	YES	YES	NA	NA	YES	YES
(33)	6/8	YES	YES	YES	YES	NA	NA	YES	YES
(34)	6/8	YES	YES	YES	YES	NA	NA	YES	YES
(35)	6/8	YES	YES	YES	YES	NA	NA	YES	YES
(36)	6/8	YES	YES	YES	YES	NA	NA	YES	YES
(37)	6/8	YES	YES	YES	YES	NA	NA	YES	YES
(38)	6/8	YES	YES	YES	YES	NA	NA	YES	YES
(39)	6/8	YES	YES	YES	YES	NA	NA	YES	YES
(40)	6/8	YES	YES	YES	YES	NA	NA	YES	YES
(41)	6/8	YES	YES	YES	YES	NA	NA	YES	YES

**TABLE 5:** Characteristics of the studies included in the systematic review (COVID-19, Ecuador, 2020–2022).

Study	Context	Study objective	Type of study	Participants	Methods	Main findings	JBI
(42)	Jilin Province, Northeast China	To evaluate the direct effects of work stress, health status and presenteeism on task performance, and further explore the mediating effects of health status and presenteeism, hoping to provide theoretical basis for improving the performance of medical staff	A cross-sectional study	4,261 medical staff	The Challenge and Hindrance-Related Self-Reported Stress scale, Short Form-8 Health Survey scale, Stanford Presenteeism Scale and Task Performance Scale	Work stress and presenteeism had a significant negative effect on task performance of medical staff, unlike health status, that had a significant positive effect on task performance. Health status and presenteeism mediated the relationship between work stress and task performance	6/8
(10)	New Zealand	To address the research gap of examining the relationship between meaningful work and dimensions of job burnout with work engagement as the mediator, especially in times of the COVID-19 pandemic	A cross-sectional study	530 social workers	The partial least squares structural equation modelling (PLS-SEM). The survey consisted of three instruments: WAMI, UWES-9 and MBI-22	Work engagement was found to have mediating effects on the relationships between meaningful work and all the dimensions of job burnout. Age does not have any moderating effect on these relationships	6/8
(43)	Netherlands	To achieve insight into COVID-care participation of surgical residents in the Netherlands, the impact of COVID-19 on the experienced quality of surgical training, and the influence on Burn out and Work Engagement compared with the non-COVID-19 period in January 2020	A cross-sectional study	317 surgical residents	Dutch questionnaire “Utrecht Burn-out Scale,” derived from the MBI, was surveyed, and also the UWES was collected	The study shows a significant impact of the first months of the COVID-19 pandemic on the surgical trainee programme. The study emphasises the need for adequate guidance of all surgical residents regarding surgical training and education	6/8
(44)	Chengdu, China	To examine whether an employee’s perceived COVID-19 crisis strength will decrease an employee’s work engagement and taking charge at work	A multi-study	258 nurses: study 1; 61 medical professionals employed in ICU	First, hypotheses were tested by conducting a time-lagged field survey of nurses who provided care to COVID-19 patients (Study 1). Next, the research question was addressed by conducting a longitudinal field experiment (Study 2) in an intensive care unit (ICU) for COVID-19 patients in critical condition. Instruments/variables: Perceived COVID-19 Crisis Strength, Work Meaningfulness, UWES-9, and Taking Charge at Work	The research demonstrates that organizations can soften the impact of this crisis on their employees by providing interventions designed to weaken perceived COVID-19 crisis strength and strengthen work meaningfulness	6/8
(45)	Wuhan, China and United Kingdom	To examine whether mindfulness may be able to neutralise the negative effects of the COVID-19 stressors on work engagement through the mediating role of sleep duration	A multi-study	97 general workers from Wuhan, China and 140 from the United Kingdom	In Study 1, a field experiment was conducted in Wuhan, China during the lockdown between 20 February 2020, and 2 March 2020, in which state mindfulness was induced by randomly assigning participants to either a daily mindfulness practice or a daily mind-wandering practice. In Study 2, in a 10-day daily diary study in the United Kingdom between 8 June 2020, and 19 June 2020, the results were replicated from Study 1 using a subjective measure of COVID-19 stressors and a daily measure of state mindfulness	Findings of the studies contribute to the employee stress and wellbeing research as well as the emerging mindfulness research in the organizational literature. As a result, mindfulness buffers the negative effect of COVID-19 stressors on work engagement mediated by sleep duration	6/8
(46)	Turkey	To explore software professionals’ mental wellbeing and work engagement and the relationships of these variables with job strain and resource-related factors in the forced home-based work setting during the COVID-19 pandemic	A cross-sectional study	321 software professionals	Survey including questions on sociodemographic characteristics, home-based work-related parameters during COVID-19, validated scales related to the participants’ mental wellbeing, work engagement, sleep quality, work-related psychosocial characteristic of job strain and decision latitude, and close-ended questions for work-life balance and physical exercise habits, was administered, all in Turkish	The results indicate that despite the negative effect of job strain, the resource-related protective factors, namely, sleep quality, decision latitude, work-life balance, and exercise predict mental wellbeing. Additionally, work engagement is predicted by job strain, sleep quality, and decision latitude	6/8
(11)	Spain	To assess the effects of the COVID-19 on the physical and mental health of non-healthcare workers. Design: Observational descriptive cross-sectional study	A cross-sectional study	1,038 non-healthcare worker (461 worked away from home and 577 workers who were working from home)	Instruments/variables: work engagement UWES-9, sense of coherence (SOC-13), and mental health (Goldberg GHQ-12)	At low levels of engagement, the percentage of distress is higher (77.9%). Low levels of sense of coherence correspond to the highest percentages of distress (86.3%). The 94.1% believe it necessary for professionals and volunteers involved in COVID-19 to receive psychological support. Low comprehensibility is mediated by the perception of stress; if the perception is low, comprehensibility is modulated by the level of significance; if it is low, it generates 95.9% of distress	6/8
(21)	Spain	To analyse the perception of COVID-19 by nurses, especially about measures, resources, and impact on their daily work. Also, to analyse these professionals’ psychosocial risks and the relationship between perception of COVID-19 and these risks	A descriptive correlational study	92 nurses	Data were collected *via* an online self-completed questionnaire during the rise of the pandemic from 29 March to 8 April, when the number of infections went from 78,797 to 146,690	There seems to be a negative and significant relationship between the information available to nurses, the measures implemented, and resources with some of their psychosocial risks, and a positive one with job satisfaction and work engagement. There is also a positive and significant relationship only between the impact of COVID-19 and their work inequality, but not for other risks	6/8
(47)	Spain	To assess psychological distress (PD) of occupational healthcare workers and its relationship with their work engagement (WE) and work environment characteristics	A cross-sectional study	499 nurses and physicians	Variables included demographic data, work environment characteristics, UWES-9, and GHQ-12	A total of 65.53% of the occupational healthcare professionals who participated had PD. No significant differences were found between physicians and nurses. However, PD was higher among women and public sector workers. Variables that facilitate developing PD were work stress, workload, the presence of labour conflict, and less job satisfaction	6/8
(29)	Punjab, Pakistan	To identify the dominance of psychosocial job demands and job resources on the wellbeing of nurses with an indirect effect on psychological health factors	A cross-sectional study	208 nurses	Time-lag strategy to collect data at the start of pandemic (Time 1) and then again 3 months later (Time 2)	Three stages were achieved through this analytic study on the nurses’ samples to determine the predictive abilities for the quality of the psychosocial work environment model. And as a result, from partial to full mediation, stress and eustress significantly impact the psychosocial work environment of nurses	6/8
(30)	Wuhan, Hubei Province, China	To clarify both the potential influencing factors and the current status of front-line nurses’ work engagement, and thus provide a References for targeted interventions	A cross-sectional study	1,040 nurses	A large sample survey was conducted at the end of February 2020 in a designated hospital treating coronavirus disease 2019 patients in Wuhan, the capital of Hubei Province, in China	The final model interpreted 27.3% of the variance, of which each block could explain 11.7%, 10.3% and 7.9% R2 changes including sociodemographic characteristics, stress and workload, respectively. Work engagement was negatively correlated with stress and workload	6/8
(15)	Spain	To assess the mediating role of work engagement in the direct impact of emotional intelligence on healthcare professionals’ work performance	A cross-sectional study	1,549 healthcare workers (62.1% women; mean age 36.51 years) (26.9% nurses)	A total of 1,549 healthcare workers (62.1% women; mean age 36.51 years) filled the Wong and Law Emotional Intelligence Scale, the UWES, and the Individual Work Performance Questionnaire	The results demonstrated in this investigation evidence the significant direct effect of emotional intelligence toward individual work performance, as well as the mediating involvement of engagement, in a sample of Spanish healthcare professionals considering the three constructs of engagement, vigour, which emerged over dedication, and absorption as the most decisive engagement dimension	6/8
(5)	Switzerland	To examine the impact of work modalities, job-related, relational, and organizational climate variables on employees’ engagement, exhaustion, and perceived performance both before and during the forced teleworking period	A cross-sectional study	1,373 Public Employees (19–60 years)	Keeping in mind the pandemic and telework conditions, the survey method was a quantitative methodology, which was deemed to be most suitable for collecting data from participants. Data were collected from a single Swiss Cantonal administration located in the French speaking part of the country	Results show that while the forced telework period positively influenced employees’ work autonomy and work–life balance, it negatively influenced their degree of collaboration and perceived job strain but did not affect their engagement levels	6/8
(31)	Netherlands	To study burnout and its association with work engagement and resilience among Dutch intensivists in the aftermath of the COVID-19 crisis	A cross-sectional study	162 intensivists	The questionnaire consisted of questions on personal and work-related characteristics and validated questionnaires: the MBI, the UWES, and the Resilience Evaluation Scale	A raised risk for burnout was found among Dutch intensivists in the wake of the COVID-19 crisis. However, this was still low compared to other countries. Work engagement was found to be high. Burnout was inversely related to, but not fully explained by, resilience and work engagement	6/8
(32)	Pakistan	To assess how individuals perceive WFM, which is affecting their daily work routine in the pandemic	A cross-sectional study	Teachers from government schools in Pakistan, mean age 37.2	The multilevel modelling (MLM) approach was applied for analysing the data to model the relationship between day-level social media misinformation, perceived COVID-19 threat, anxiety, social media fatigue, and work engagement	Findings revealed that misinformation and COVID-19 threat increase anxiety and social media fatigue, resulting in a lower level of work engagement. This study also found that resilience as a coping mechanism reduces the adverse effects of anxiety on work engagement	6/8
(33)	Italy	To investigate the impact that family-work conflict, social isolation, distracting environment, job autonomy, and self-leadership have on employees’ productivity, work engagement, and stress experienced when WFH during the pandemic	A cross-sectional study	A total of 209; mean age 49.81; minimum: 25; maximum: 65	This cross-sectional study analysed data collected through an online questionnaire completed by 209 employees WFH during the pandemic. The assumptions were tested using hierarchical linear regression	Family-work conflict and social isolation were negatively related to WFH stress, which was not affected by autonomy and self-leadership. Individual and work-related aspects both hinder and facilitate WFH during the COVID-19 outbreak	6/8
(34)	Germany	To investigate the stress perception of German outpatient nurses during the COVID-19 pandemic. The aim was to determine associations between their pandemic related stress and variables such as sleep quality, work engagement, pandemic-related worries and concerns	A cross-sectional study	166 nurses	An online questionnaire study was conducted among German outpatient nurses from outpatient care services	Pandemic-related stress proved to be a predictor of poorer quality of sleep among outpatient nurses (H1) and Pandemic-related stress proved to be a predictor of lower work engagement among outpatient nurses (H2). Pandemic-related concerns and worries were not positively related to higher stress experience among outpatient nurses (H3)	6/8
(35)	Poland	To examine how different forms of work affect employee behaviour	A cross-sectional study	544 participants	This study applies work engagement (the key construct in organisational psychology) as the dependent variable and considers its determinants in the form of stress factors and attitudes toward remote work. UWES-9, Stress Management Standards, and Attitudes toward Remote Work were used	The obtained results indicate that there were no significant differences between groups in terms of the intensity of work engagement. For on-site workers, the most important factors were control and role definition	6/8
(36)	Zagazig, Egypt	To assess the mattering perception, feelings of burnout and work engagement amongst nurses during the coronavirus outbreak	A cross-sectional study	280 nurses	A self-administered questionnaire containing four parts: characteristics, mattering at Work Scale, Burnout scale, and Engagement scale	There was a statistically significant positive correlation between engagement and mattering perception. However, there was a statistically significant negative correlation between burning out with engagement and mattering	6/8
(37)	United States	To understand how nurses’ work engagement has been affected by COVID-19	A cross-sectional study	107 nurses	A descriptive, cross-sectional design was used. A survey plus the Fear of COVID-19 scale, the Utrecht Work Engagement Scale, and three open-ended questions were used	The type of education significantly correlated with engagement scores, with in-service education having the highest scores. Nurses continue to leave the profession because of high patient census and acuity and inadequate staffing	6/8
(38)	Ecuador	To find the relationship between work environment factors and work engagement among the Ecuadorian general population during the first phase of the COVID-19 pandemic to assess their levels of psychological distress	A cross-sectional study	2,161 participants	Sociodemographic and work environment data, work engagement (UWES-9 scale) scores, and General Health Questionnaire (GHQ-12) scores were collected	The factors that, to a large extent (70.2%), predicted the development of PD during the first phase of the COVID-19 pandemic in Ecuador were being a woman and having low levels of the vigour work engagement dimension, high work stress, and low job satisfaction	6/8
(39)	United Kingdom (UK)	To describe the work engagement perceived by UK workers during the COVID-19 pandemic	A cross-sectional study	1,085 participants	Data were collected using an online questionnaire and the UWES-9	Participants with lower satisfaction (21.8%) gave significantly low or very low UWES-9 scores in 58.5% of the cases. Greater work engagement was obtained with more resources and less conflict, risk, and stress. In cases where there had been contact with COVID-19, this was associated with slightly lower levels of work engagement	6/8
(40)	Mainland China	To clarify the mediating mechanism and boundary conditions between risk perception and employee work engagement, explore the causal mechanism of work engagement, and provide practical organisational guidance for maintaining employee work engagement in response to the COVID-19 epidemic	A cross-sectional study	285 participants	Regression analysis and bootstrap tests were conducted on SPSS and AMOS to verify the relevant hypotheses	It is demonstrated that the moderating effects of employee psychological resilience are all positive on mediating effects of risk perception, anxiety and work engagement. For employees with high psychological resilience, the mediating effect of risk perception on work engagement is stronger through anxiety	6/8
(41)	Mexico	To identify the presence of high levels of work engagement and burnout in COVID-19 response teams (RT) during the COVID-19 pandemic in a secondary care level	A cross-sectional study	156 participants	UWES-9 and the MBI-HSS scales	High levels of work engagement were identified in 55.1% of the COVID-19 RT members, while the high levels of burnout were 3.2%. The prevalence of work engagement was higher than that of burnout, but this did not imply protection against exhaustion	6/8

WAMI, work and meaning inventory; UWES-9, Utrecht work engagement scale; MBI-22, Maslach Burnout Index.

## Results

**FIGURE 1:**
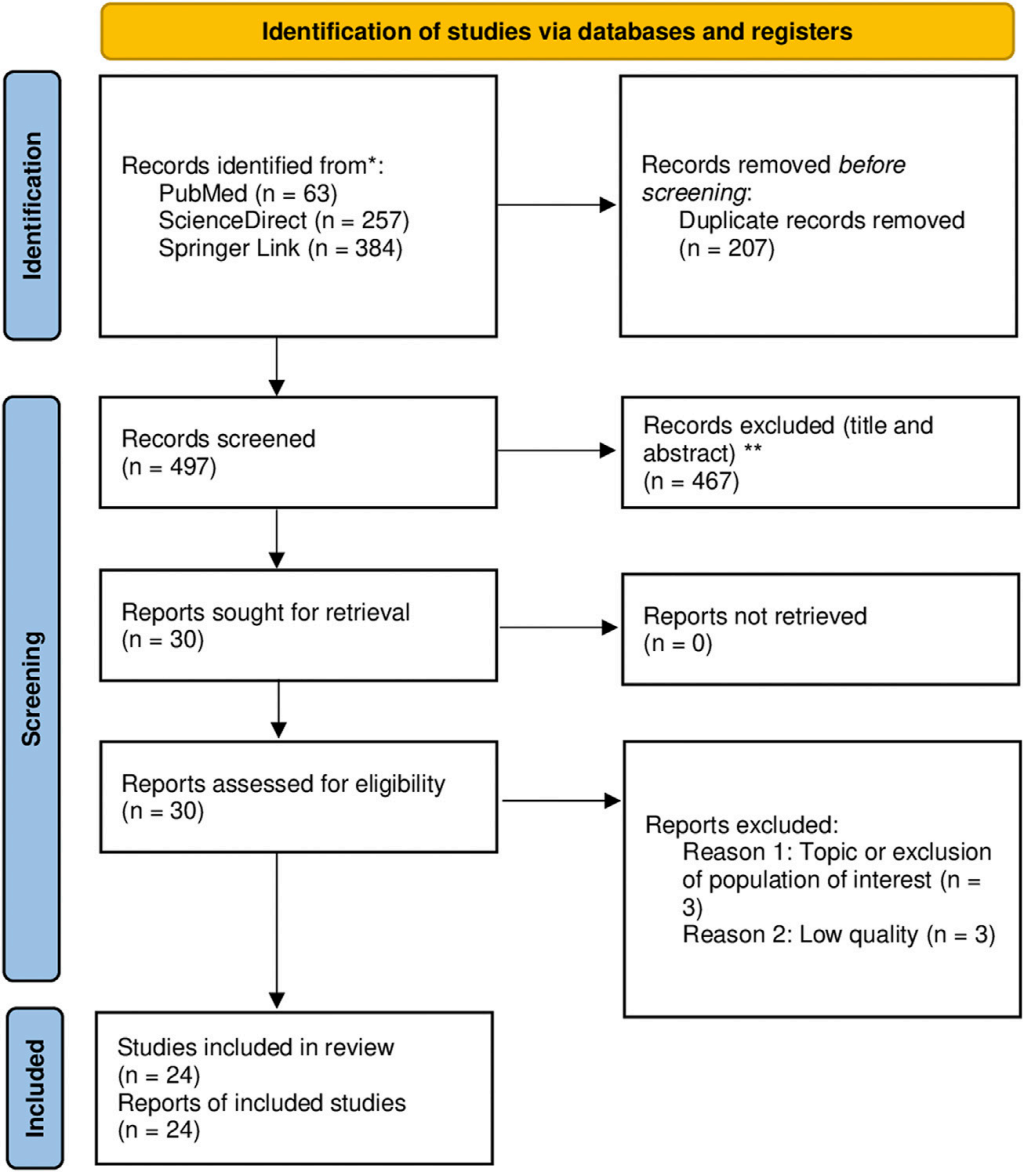
PRISMA 2020 flow diagram (25) (COVID-19, Ecuador, 2020–2022).

The initial search strategies identified a total of 704 references, which were screened according to the topic of this review. A total of 24 studies (5, 10, 11, 15, 28–47) were finally selected (Figure 1).

All the analysed studies were quantitative. Of the 24 studies, 18 were conducted in the first phase of the pandemic, i.e., from December 2019 to June 2020, and 6 were conducted based on data collected until August 2021. There were 13 of the 24 studies focused on healthcare workers, with 9,469 participants divided as follows: 6,707 were women, representing 71%. In addition, of these 13 studies, 9 were specifically focused on nurses. The remaining 11 studies were focused on general workers, including social workers, software developers, teachers, and service personnel. In 1 of the 11 studies, there were 1,038 participants, but no data by sex was offered. In the other 10 studies, there were 7,828 participants in total, of which 4,539 were women, representing 58% of the samples. Of the 24 articles, 11 were conducted in Europe and 8 in Asia, 1 in Africa, 1 in Oceania, and 3 in the Americas.

## Discussion

The aim of this review was to assess the relationship between psychological distress (stress or burnout) and work engagement during the COVID-19 pandemic. To do so, the levels of stress, burnout, and work engagement were analysed in the 24 selected studies, and other factors were added that also influence work engagement.

All the articles carried out their studies with full-time employees over 18 years of age, but it is important to highlight the participation of women, with 71% in the group of healthcare staff and more than 50% in the case of general workers. In the case of healthcare personnel, the cause could be that most of the articles focused on nurses, a position in which women stand out.

With regard to healthcare workers, a study conducted in China among 258 nurses and 61 physicians (30) analysed the negative impact of the COVID-19 pandemic and work commitment, and the results showed that they were negatively correlated, but that if the necessary training was provided, the perception of risk decreased and work commitment increased. In another study in the same country with 1,040 nurses, it was also found that work engagement was negatively correlated with stress and burnout, and that women had lower levels of work engagement than men, with 43.02 and 47.25, respectively (35), as in the study by Jia et al. (28). Although these are values of engagement considered at a moderate level, in this study the factors that influenced to obtain better levels of work engagement were: being between 31 and 40 years old, being married, having more than two children, having a master’s degree, among others. Job stress and presenteeism had a significant negative impact on task performance, which is related to two of the three dimensions of work engagement, namely absorption and dedication. In a study also conducted in China, with 4,261 physician participants, moderate levels of work engagements were found (28). The factors that influenced these results were being between 41 and 50 years old, being married, and having university education. In these studies there was a negative correlation between psychological distress and work engagement, that certain socio-demographic factors could influence, such as sex, being married, age, or years of experience, and that the assistance and training that the organisation can offer were also determinants in reducing the perception of risk and, therefore, increasing work engagement.

Directing our vision towards healthcare workers, in a study conducted in the Netherlands on burnout and work engagement, no significant differences were found between men and women (29). However, residents who were on the COVID-19 area had higher levels of burnout than those who were not assigned to the COVID-19 patients, with 16% and 7.6%, respectively. This may be due to working conditions (workload and concurrence of negative events) and the emotional impact that the pandemic had, especially in the initial stages of it (48). Regarding work engagement, residents who were assigned to intensive care patients evidenced a higher impact of the COVID-19 pandemic on their daily routine. Yet, no differences were observed before and after the pandemic for residents who did not care for COVID-19 patients, possibly due to a specific training programme for surgery and strict hygiene conditions, that may lead to the high level of enthusiasm among residents in that country (29). In Spain, a study on 92 nurses found that, despite the pandemic and its impact, work engagement was moderate to high, perhaps because this group of professionals was aware of the importance of their work (47). In the same country, the study carried out on 499 nursing staff participants revealed that work engagement was 34.80 and the participants with low levels of engagement had high levels of psychological distress 76.7% (33), which indicated a negative correlation between these two variables. Meanwhile, in Germany, in a study on 166 nurses, it was found that half of the participants had low levels of stress, during the COVID-19 pandemic (39). It is worth mentioning that the data in this study were taken up to May 2021, the second year of the pandemic, which may partly explain the high level of work engagement. Also, in a Dutch study on 162 intensivists, 98 men and 64 women, burnout levels were 5.1% and 12.5%, respectively, while work engagement was assessed at low, moderate, and high levels, with men reporting 43.9% and women 31%. Although burnout values were low, women had a higher level, and women’s work engagement scores were better than men’s (36). In the Netherlands and Germany, similar levels were found to another study conducted in nurses in Egypt, with low levels of psychological distress were found, and moderate to high work engagement, while in Spain the levels of distress were higher with respect to the results of these countries, and work engagement was moderate. It could be deduced then that the organisational systems of each country and the allocated resources in each hospital can also influence psychological distress and, therefore, the level of work engagement, in addition to other factors such as sex, age, or being married.

In Mexico, there was a prevalence of high levels of work engagement, higher than levels of burnout, but this did not imply protection against burnout (46). The results regarding work engagement were very similar to those found in the United States (42), which may be explained by the fact that these two studies were conducted in the second year of the pandemic, by which time more was already known about how to cope with the pandemic.

With regard to general workers, a study conducted in Ecuador on 2,161 general workers determined that 62.72% of the population had psychological distress, with women having higher levels (69.1% vs. 55%) (43). As shown by studies on healthcare workers, sex, age, having children or not, level of education, and being married are also factors that influence the results (49). In another study conducted in Spain on non-healthcare workers, it was found that at low levels of engagement there were higher percentages of psychological distress (77.9%), and this same trend was observed both in the group of workers who worked away from home and among those who worked from home (11). Following this, a study conducted in the UK on 1,038 general workers (44), in line with a study conducted in New Zealand on 530 social workers (10), found that work engagement was an effective predictor of reduced burnout, cynicism, and feelings of reduced professional competence.

In general, results from several countries show that psychological distress (stress or burnout) does have a significant negative effect on work engagement. However, other factors such as presenteeism may also play a role (28). On its part, meaningful work is another factor that can influence work engagement (10) and understanding the needs of healthcare workers during a pandemic is critical to attracting and retaining them (42). On the other hand, mindfulness and the quantity and quality of sleep are also factors to be considered, as indicated by a study conducted in Wuhan, China and replicated in the UK with general workers. The results revealed that there was a positive relationship between the amount of sleep and work engagement (31). Emotional intelligence is another factor to analyse, and a study conducted with Spanish workers indicated that there was evidence of a significant direct effect of emotional intelligence on individual job performance, as well as a mediating effect regarding work engagement (15).

The present study offers a number of limitations. Firstly, it should be noted that articles that were only written in English or Spanish were included, which may have left out articles that met the rest of the inclusion criteria. Secondly, it is important to stress that 13 of the 24 articles focused on healthcare workers and the rest on general workers, yet working in administrative or service areas. Therefore, the results cannot be extrapolated to professions in other sectors such as manufacturing, construction, food, etc. The generalisation of the results of this review should be considered with caution, as the main data come from studies in different countries, with different instruments and methodologies.

### Conclusion

Based on the articles reviewed, it can be concluded that psychological distress or stress levels do have a significant impact on work engagement, as does burnout. However, there are other influencing factors such as presenteeism, meaningful work, mindfulness, and even emotional intelligence. On the other hand, with respect to healthcare workers and despite the COVID-19 pandemic, the results concerning work engagement have been moderate to high, while the results regarding psychological distress (stress or burnout) do differ among countries.

To minimize stress levels and encourage work engagement, organizations must take actions to ensure safety in the work environment, for example, promoting strategies that enable employees to understand their contribution to the goals of the organisation, their impact on the care and wellbeing of others, and their own personal growth. In addition, the ability of institutions to allocate the necessary resources and information to cope with a health crisis can be crucial to ensure that despite the heavy workload that healthcare workers have in such situations, specifically nurses and healthcare professionals, satisfaction can be derived from what they do and, at the same time, this may become a protective factor against physical and psychological harm.
